# Early-Onset Rectosigmoid Junction Cancer Treated With Laparoscopic Anterior Resection and Indocyanine Green-Guided Anastomosis Within an Enhanced Recovery After Surgery (ERAS) Protocol: A Case Report

**DOI:** 10.7759/cureus.107626

**Published:** 2026-04-23

**Authors:** Eduardo D Cruz Mendoza, Roberto A Nuñez González, Ricardo Sordo Mejía, Andrea Paniagua Quiroga, José A Morales Mendoza

**Affiliations:** 1 General Surgery, Hospital de Especialidades Instituto Mexicano del Seguro Social (IMSS), Puebla, MEX; 2 Coloproctology, Hospital Ángeles Puebla, Puebla, MEX; 3 Coloproctology, Hospital Puebla, Puebla, MEX; 4 Anesthesiology, Hospital Ángeles Puebla, Puebla, MEX; 5 Medicine, Instituto de Estudios Superiores de Chiapas (IESCH), Tuxtla Gutiérrez, MEX

**Keywords:** colonoscopy, colorectal neoplasms, enhanced recovery after surgery (eras), genetic testing, laparoscopy

## Abstract

Colorectal cancer (CRC) diagnosed in individuals under 50 years of age is referred to as early-onset CRC. In recent decades, a significant increase in both its incidence and mortality has been observed. These patients are frequently diagnosed at advanced stages, which is associated with a higher likelihood of presenting with advanced disease at the time of diagnosis.

We present the clinical case of a 35-year-old male patient with colon cancer. According to the American Joint Committee on Cancer(AJCC) TNM staging system, the clinical stage corresponded to stage I (T2N0M0). No molecular testing was performed due to the early clinical stage. His perioperative management included the implementation of an Enhanced Recovery After Surgery (ERAS) protocol, aimed at optimizing postoperative recovery. In addition, intraoperative assessment of intestinal perfusion using indocyanine green (ICG) fluorescence facilitated a minimally invasive surgical approach. The case is complemented by a literature review on early-onset colon cancer, with the aim of highlighting age-related differences in CRC incidence and analyzing potential factors contributing to the rising incidence and mortality in younger patients.

## Introduction

Colorectal cancer (CRC) represents one of the most significant global public health challenges and remains among the leading causes of cancer-related mortality worldwide. Traditionally, it has been considered a disease predominantly affecting individuals over 50 years of age. However, over recent decades, a sustained increase in CRC incidence among younger adults has been documented, a phenomenon termed early-onset CRC. This designation refers to cases diagnosed before the age of 50 and constitutes a distinct clinical subtype with unique epidemiological and biological characteristics. Multiple studies have demonstrated that its incidence continues to rise, contrasting with the declining trend observed in older populations, largely attributable to screening programs [1].

Early-onset CRC is often diagnosed at more advanced stages and frequently exhibits specific histopathological features, including mucinous histology, increased lymphovascular invasion, and a predilection for distal colon or rectal locations. These characteristics, combined with diagnostic delays due to low clinical suspicion in younger patients, complicate management and adversely impact prognosis [2].

Genetic predisposition is particularly relevant in this population. Approximately 16-25% of patients with early-onset CRC harbor a hereditary cancer predisposition syndrome, with Lynch syndrome and familial adenomatous polyposis being the most notable. In sporadic early-onset CRC, recent evidence shows a predominance of tumors with an immunologically "cold" tumor microenvironment, characterized by microsatellite stability and low lymphocytic infiltration, which is associated with a limited response to immunotherapy. At the molecular level, these tumors predominantly cluster into CMS2 and CMS3 subtypes, with the activation of the WNT/MYC pathways in CMS2 and metabolic alterations associated with KRAS mutations in CMS3, suggesting a more epithelial and less immunogenic tumor biology. Both are associated with germline mutations affecting DNA repair mechanisms or intestinal epithelial growth regulation. Identification of these alterations has significant clinical implications, as it enables tailored surveillance, informs surgical planning, and facilitates targeted screening of first-degree relatives [3].

For localized CRC, oncologic surgical resection remains the cornerstone of curative treatment. Surgery must adhere to strict oncologic principles, including the resection of the affected colonic or rectal segment with adequate margins and appropriate regional lymphadenectomy. In recent years, perioperative management has evolved with the adoption of Enhanced Recovery After Surgery (ERAS) protocols, which integrate evidence-based interventions to optimize the physiological response to surgical stress. These programs encompass multimodal preoperative preparation, standardized anesthesia, early mobilization, and nutritional optimization and have been shown to reduce postoperative complications, shorten hospital stays, and accelerate functional recovery [4].

Case reports in younger patients are particularly informative, providing insight into clinical presentation, genetic implications, and therapeutic strategies in this emerging population. Here, we present the case of a patient with early-onset CRC who underwent surgical treatment following an ERAS protocol. This report emphasizes the importance of a comprehensive, multidisciplinary approach and highlights the use of indocyanine green (ICG) fluorescence imaging with near-infrared visualization for ureteral identification and delineation to minimize the risk of ureteral injury. Furthermore, this technique enables the intraoperative assessment of intestinal perfusion, confirming the adequate vascularization of both the resected segment and anastomotic margins. The procedure was complemented by intraoperative colonoscopy to directly evaluate anastomotic integrity, perfusion, and hemostasis, facilitating the early detection of potential defects or leaks [5,6].

## Case presentation

A 35-year-old male patient with no prior medical or surgical history presented to the coloproctology clinic with a three-month history of lower gastrointestinal bleeding associated with bowel movements, without additional symptoms. There was no family history of gastrointestinal malignancies.

**Figure 1:**
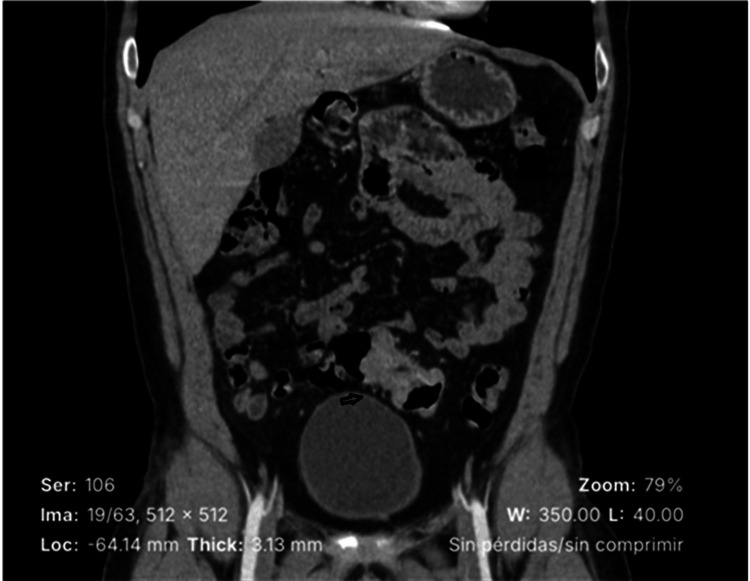
CT of the abdomen and pelvis with intravenous contrast CT demonstrates circumferential wall thickening of the sigmoid colon (arrow). CT: computed tomography

Computed tomography (CT) of the abdomen and pelvis revealed concentric wall thickening of the sigmoid colon, resulting in significant luminal narrowing over an approximately 3 cm segment, along with a pericolonic lymph node located at the mesenteric border adjacent to the lesion (Figure 1).

A thoracoabdominopelvic CT scan showed no evidence of metastatic disease.

**Figure 2:**
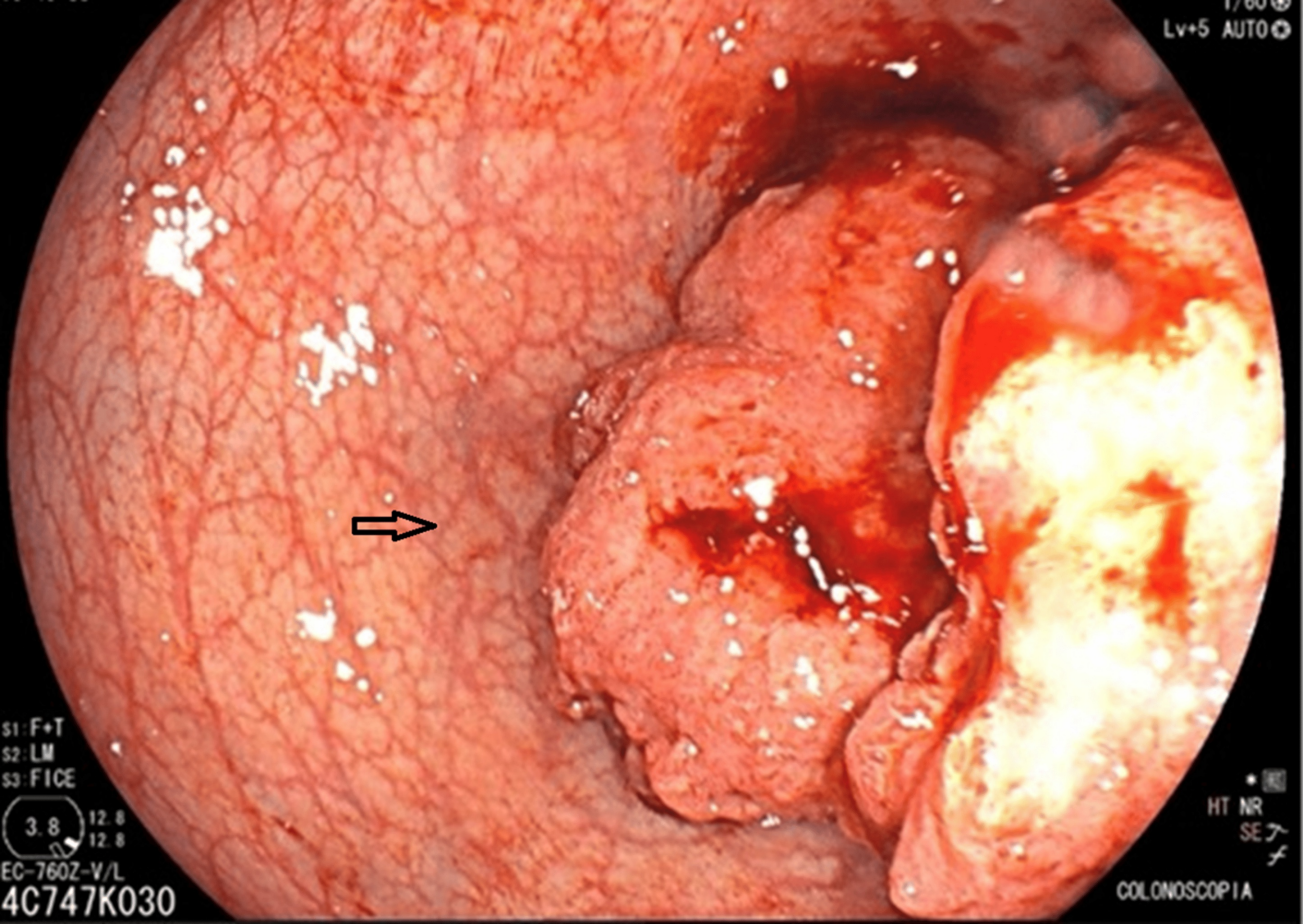
Colonoscopy Colonoscopy image demonstrating a tumor at the rectosigmoid junction with ulcerated areas on its surface (arrow).

The carcinoembryonic antigen (CEA) level was 1.82 ng/mL. Subsequently, the patient underwent colonoscopy, which revealed an exophytic lesion at the rectosigmoid junction involving approximately 40% of the circumference. The lesion was irregular, with an ulcerated surface and areas of bleeding, and showed loss of the normal glandular pattern (Figure 2).

Two pedunculated polyps were identified 3 cm from the lesion and were removed by endoscopic mucosal resection (EMR).

**Figure 3:**
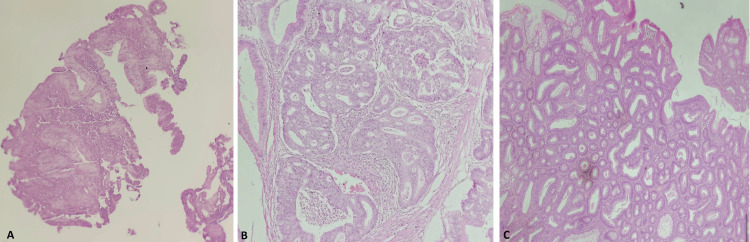
Histopathological report of the colonoscopy biopsy Histopathology report using hematoxylin and eosin staining at 10× magnification revealed a moderately differentiated invasive adenocarcinoma (A). Polyp analysis demonstrated tubular adenomas with high-grade dysplasia (B and C).

Histopathological examination of the biopsy revealed a moderately differentiated, invasive adenocarcinoma with an intestinal phenotype. Analysis of the resected polyps demonstrated three tubulovillous adenomas with high-grade dysplasia (Figure 3).

The patient subsequently underwent a laparoscopic anterior resection with mechanical anastomosis guided by ICG fluorescence, following an ERAS protocol (Table 1).

**Table 1:** Description of the ERAS protocol implemented in this patient This table describes the implementation of the ERAS protocol, including the preoperative, intraoperative, and postoperative phases. ERAS: Enhanced Recovery After Surgery

ERAS phase	Interventions in the patient
Preoperative	Patient education regarding the procedure and ERAS protocol. Nutritional optimization with a high-calorie, high-protein diet. Prehabilitation: daily aerobic exercise (30 minutes), strength training, and respiratory exercises. Cessation of tobacco and alcohol use. Hospital admission 24 hours prior to surgery. Clear liquid diet. Bowel preparation with sodium picosulfate. Oral antibiotic prophylaxis (metronidazole + ciprofloxacin). Preoperative carbohydrate loading with maltodextrin. Intravenous hydration with Hartmann's solution. Thromboprophylaxis with enoxaparin and graduated compression stockings
Intraoperative	Minimally invasive (laparoscopic) surgery. Intravenous antibiotic prophylaxis (metronidazole + ceftriaxone) administered 30 minutes prior to incision. Mechanical anastomosis
Postoperative	Early mobilization. Early initiation of oral intake. Multimodal pain control. Monitoring of bowel function. Hospital discharge at 72 hours without complications

Preoperative optimization measures were implemented starting two weeks prior to surgery. Nutritional supplementation with a high-calorie, high-protein formula was prescribed, along with a prehabilitation program that included daily aerobic exercise (30 minutes), strength training, and respiratory exercises. Alcohol and tobacco use were discontinued to improve functional reserve and reduce the risk of postoperative complications.

The patient was admitted one day prior to the procedure, continuing the ERAS protocol. Clear liquid diet and bowel preparation with sodium picosulfate were initiated, complemented by intravenous hydration with Hartmann's solution. Combined oral antibiotic prophylaxis with metronidazole and ciprofloxacin was administered, and preoperative carbohydrate loading with maltodextrin was performed. Thromboprophylaxis with enoxaparin and graduated compression stockings were also implemented.

During the immediate preoperative period, intravenous antibiotic prophylaxis with metronidazole and ceftriaxone was administered 30 minutes prior to surgical incision.

**Figure 4:**
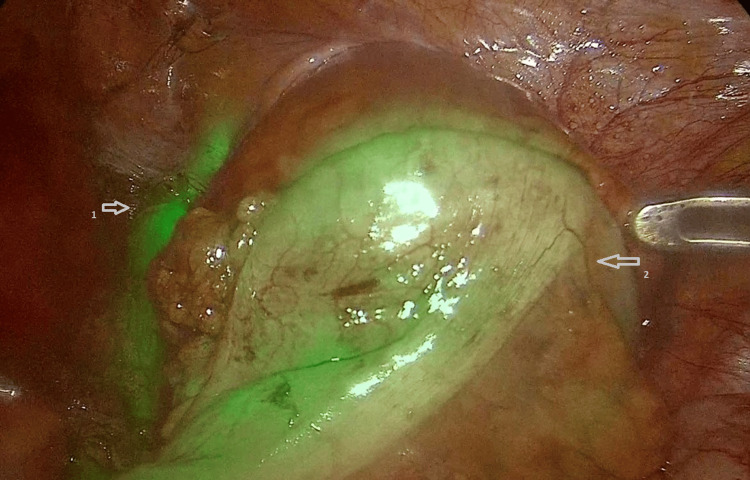
Colorectal anastomosis Intraoperative visualization using indocyanine green fluorescence, demonstrating ureteral identification (arrow 1) and adequate intestinal perfusion at the anastomotic site (arrow 2).

Minimally invasive surgery was performed using a laparoscopic approach. Intraoperatively, ICG was used for ureteral identification and the assessment of intestinal perfusion. For ureteral identification, ICG was administered through a pre-placed open-ended catheter at the beginning of the surgical procedure. For the assessment of intestinal perfusion, ICG was administered intravenously two minutes prior to colonic transection in order to ensure well-perfused margins for anastomosis. The dose was calculated at 0.2 mg/kg and diluted in 20 mL of normal saline for both applications (Figure 4).

**Figure 5:**
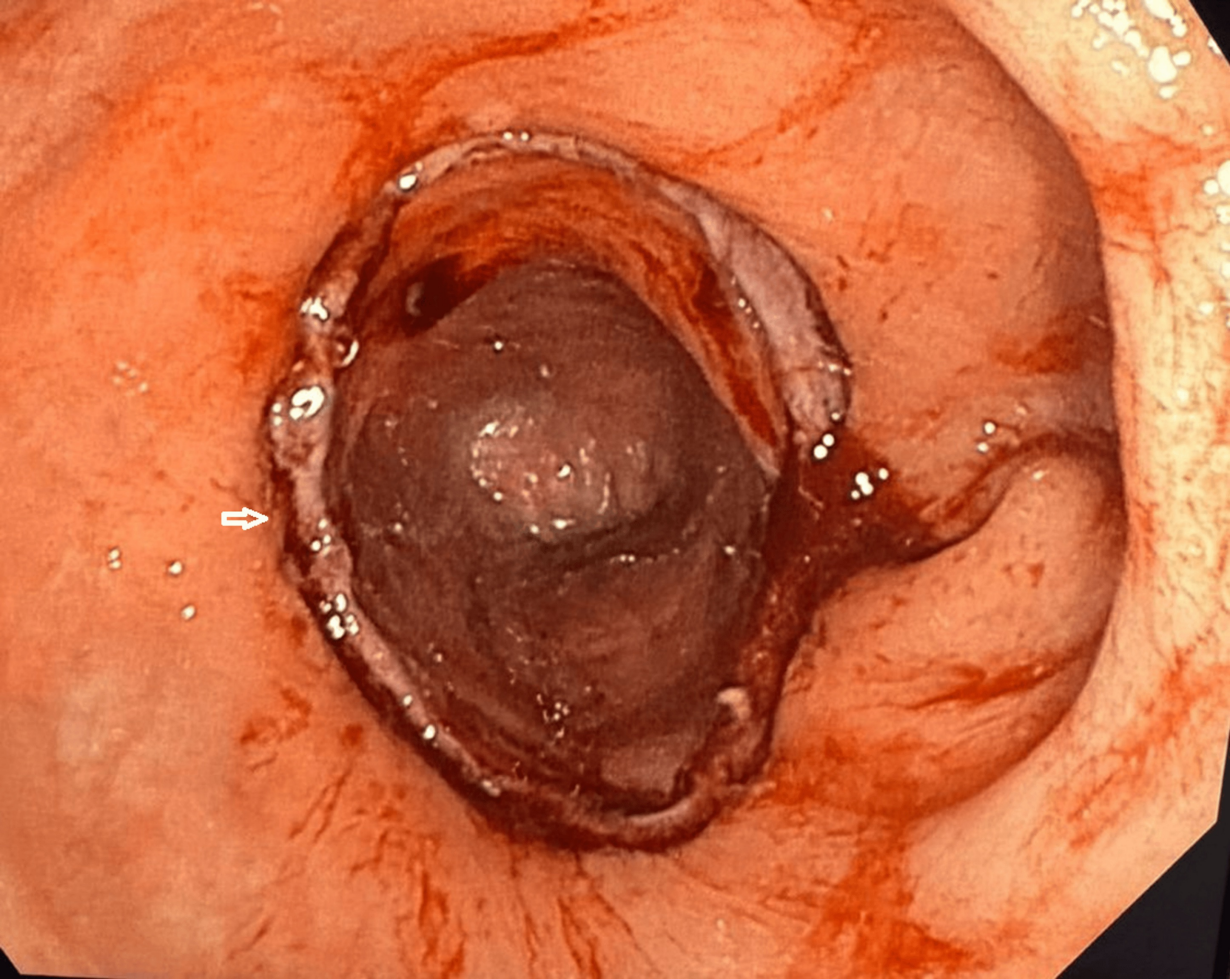
Intraoperative colonoscopy Assessment by intraoperative colonoscopy showing an intact mechanical anastomosis with adequate mucosal perfusion, no active bleeding, and no findings suggestive of a leak (arrow).

Subsequently, an intraoperative colonoscopy was performed to assess the integrity and viability of the anastomosis (Figure 5).

As part of the ERAS protocol, a liquid diet was initiated on the same day of the procedure and was well tolerated. The patient passed flatus and had bowel movements and was discharged 72 hours postoperatively in good general condition and without complications. A follow-up evaluation was performed at 10 days for the removal of laparoscopic port-site sutures and again at 30 days, with no complications observed. The patient was referred to the medical oncology service, where it was determined that adjuvant therapy was not indicated based on the clinical stage.

**Figure 6:**
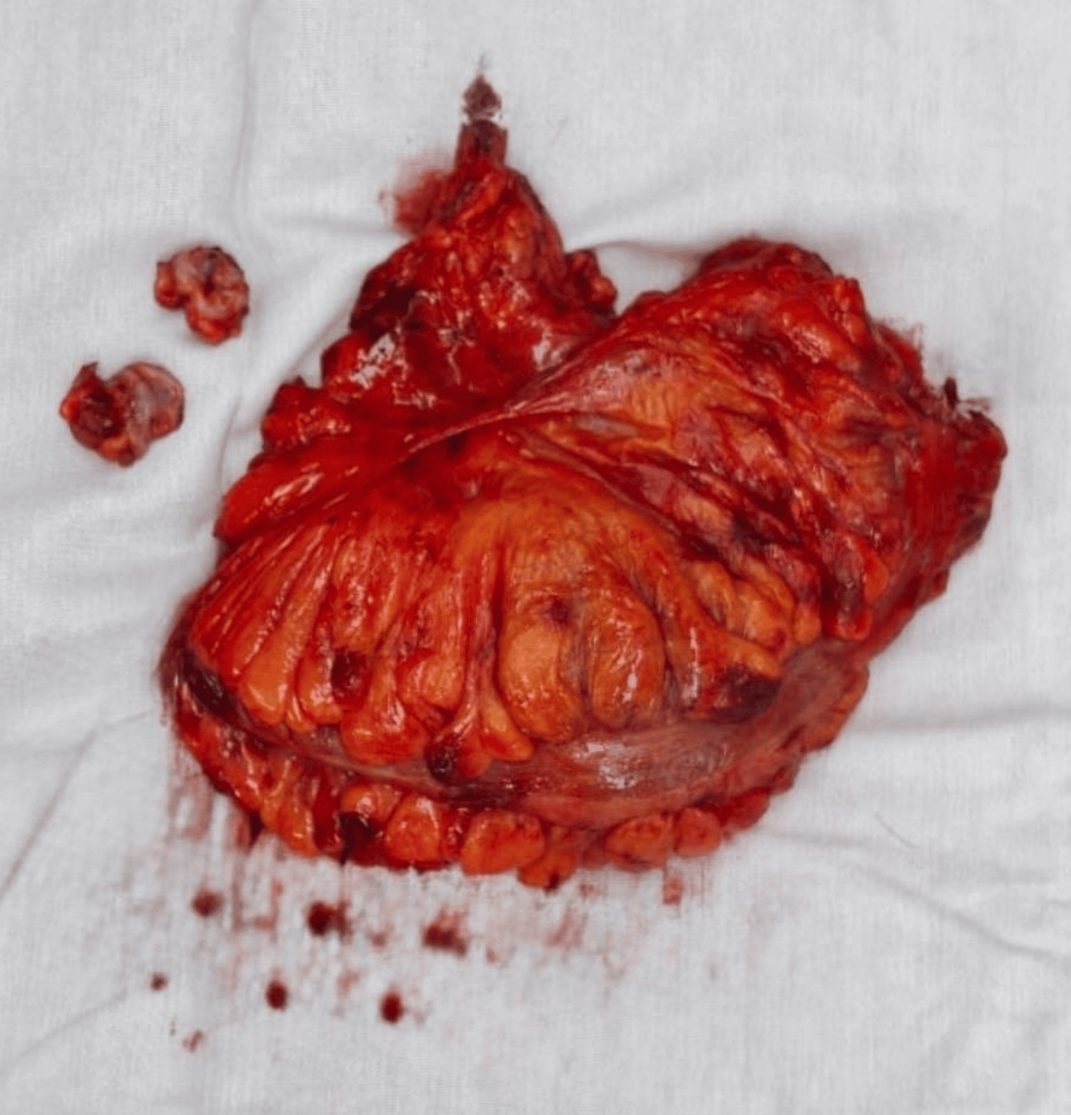
Pathological specimen The pathological specimen demonstrates a resected sigmoid colon and anastomotic rings.

Histopathological examination of the surgical specimen revealed an ulcerated, moderately differentiated adenocarcinoma of the rectosigmoid junction measuring 4 × 3 cm, with focal lymphovascular invasion. Surgical margins were free of tumor, and 21 lymph nodes were harvested, showing mixed hyperplasia without evidence of malignant cells. These findings meet the quality standards for an adequate oncologic resection in CRC. According to the American Joint Committee on Cancer (AJCC) TNM staging system, the pathological stage was I (T2N0M0) (Figure 6).

## Discussion

CRC can be asymptomatic; however, it may occasionally present with alarm symptoms such as rectal bleeding. In younger patients, the most common presentations include hematochezia, anemia, and weight loss. Adenomatous precursor lesions are less frequent in this population, and tumors are more often located distal to the splenic flexure or in the rectum. The literature reports diagnostic delays ranging from weeks to years, likely due to low clinical suspicion in this age group, contributing to detection at advanced stages or with metastatic disease [7].

The global increase in early-onset CRC has been associated with environmental and lifestyle factors, including excessive alcohol consumption, obesity, diabetes, and sedentary behavior. Screening colonoscopies have substantially reduced both the incidence and mortality of CRC over recent decades; nevertheless, a sustained increase in incidence has been observed among individuals under 50 years of age over the past 30 years [8].

From a surgical standpoint, laparoscopic anterior resection is currently the standard of care for tumors localized to the sigmoid colon and upper rectum, providing oncologic outcomes equivalent to open surgery while offering additional benefits such as reduced postoperative pain, shorter hospital stay, and faster functional recovery. In this context, intraoperative assessment of intestinal perfusion using ICG fluorescence has emerged as a valuable tool to reduce the risk of anastomotic leak, one of the most feared complications in colorectal surgery. ICG allows objective, real-time evaluation of tissue perfusion, optimizing the selection of the transection site and the viability of the anastomosis [9].

Furthermore, the implementation of ERAS protocols has revolutionized perioperative management in colorectal surgery. This approach includes perioperative optimization, minimally invasive techniques, adequate pain control with reduced opioid use, early mobilization, and early resumption of oral intake [10]. From the patient's perspective, the recovery process was well tolerated, highlighting early return of bowel function, minimal postoperative pain, and a rapid reintegration into daily activities, which contributed to a high level of satisfaction with the care received.

## Conclusions

The present case report, together with the available literature, suggests that the combination of a laparoscopic approach guided by ICG within an ERAS protocol may be associated with reduced complications, shorter hospital stay, and improved overall patient recovery, without apparent detriment to oncologic outcomes.

In the present case, the combined application of a minimally invasive technique, intraoperative perfusion assessment using ICG, and an ERAS protocol demonstrates the feasibility of a comprehensive and contemporary approach to the management of early-onset CRC. This strategy not only aligns with oncologic principles but also prioritizes functional recovery and complication reduction, highlighting the potential benefits of this approach, particularly in younger patients.

Finally, the increasing incidence of CRC in young adults underscores the importance of strengthening early detection strategies and maintaining a high index of clinical suspicion for suggestive symptoms. Furthermore, the integration of technologies such as ICG fluorescence and the standardization of ERAS protocols may contribute to improved surgical outcomes and overall quality of care in this patient population.
